# Dr. Samuel Potter

**Published:** 1897-07

**Authors:** 


Dr. Samuel Potter, of Lancaster, died at his residence in that village Saturday, June 12, 1897, aged 83 years. He had been a resident of Erie County for over fifty years, during the larger portion of which time he had been engaged in the practice of his profession, from which, however, he retired a few years ago. He was a public-spirited citizen and a man of great popularity. Two daughters survive him, Mrs. F. E. Furman, of Rochester, and Mrs. Lewis Eaton, Jr., of Lancaster.

